# Exploring the challenges of Iranian government hospitals related to Covid-19 pandemic management: a qualitative content analysis research from the nurses perspective

**DOI:** 10.1186/s12912-022-01008-8

**Published:** 2022-08-12

**Authors:** Alireza Jabbari, Sahar Salahi, Marziye Hadian, Zahra khakdel, Elaheh Hosseini, Hojjat Sheikhbardsiri

**Affiliations:** 1 Health Services Management, Health Management and Economics Research Center, Isfahan University of Medical Sciences, Isfahan, Iran; 2 Department of Nursing, Yasuj branch, Yasuj Islamic Azad University, Yasuj, Iran; 3 Health in Disasters and Emergencies Research Center, Institute for Futures Studies in Health, Kerman University of Medical Sciences, Kerman, Iran

**Keywords:** Challenges, COVID-19 Pandemic, Nurses, Govrment Hospital

## Abstract

**Background:**

The COVID-19 pandemic poses a major threat to global public health. As a result, to prepare healthcare systems for this unprecedented threat, a coordinated worldwide response is required. This study aimed to explore the hospitals challenges related to covid-19 pandemic management from the iranian nurses perspective.

**Methods:**

This study was conducted as a qualitative content analysis in Iran. Using the purposive sampling method, data were collected through in-depth individual interviews with 35 nurse personnel. Graneheim and Lundman’s conventional content analysis methods were used to analyze the data and for the trustworthiness of the data, this study used Lincoln and Guba’s recommendations.

**Results:**

After multiple rounds of analyzing and summarizing the data and taking into consideration similarities and differences, 5 main categories and 14 subcategories created based on the results of data analysis and including1) Leadership and management 2) Service delivery management 3) Human resources management 4) Equipment and Supplies Management and 5) Economic resources management.

**Conclusion:**

Identifying the most important challenges of nursing can play an important role in improving the management of COVID-19pandemic. The analysis of the challenges by managers at local, provincial and national levels can lead to the presentation of effective solutions to address these challenges and improve the pandemic management process in the country.

## Introduction

The outbreak of natural disasters is one of the main challenges faced by countries. However, the most important thing is the way senior managers, experts, and the public deal with the challenge will determine the future state of the crisis [1]. The world is now witnessing the outbreak of the COVID-19 pandemic as an infectious disease [2]. The high mortality rate and the inability of countries to control the pandemic indicate that COVID-19 management requires comprehensive efforts in all health, technological, economic, social, political, cultural, and environmental domains [3, 4].  COVID-19 is a highly pathogenic viral infection caused by the severe acute respiratory syndrome coronavirus 2 (SARS-CoV-2) [5]. The COVID-19 pandemic reminds us that we live in a very complex and unpredictable world [6, 7]. For health care providers, effective pandemic responses require deviations from many conventional practices [7, 8]. The COVID-19 pandemic has posed several new challenges for the health system [1, 4]. Policymakers and hospitals did not have enough time to adapt to sudden changes and adjust their response, resulting in an unprecedented disruption to the global health care system [9, 10].

Medical staff including physicians and nurses are at the forefront of the fight against the coronavirus. It seems that despite the care while dealing with patients, the medical staff is more likely to develop COVID-19 in infected hospital environments and, compared to the general population, they are more involved with the disease due to more frequent exposure and more viral load in these encounters [11]. Nurses work in a new environment to help COVID-19 patients. They stand at the forefront, devoting themselves to controlling the disease, regardless of any risk [12, 13]. They work around the clock to provide ongoing care for critically ill patients, many of whom use artificial respiration and need very complex care [14, 15]. Nurses have been also actively involved in hospital leadership and management activities, such as mobilizing hospital resources to confront the epidemic [16]. Thus, they face many challenges because of their high workload and workplace pressure [11, 17].

## Litrature review

In Italy, Bambi et al. (2020) investigated the new issues in nursing management during the COVID-19 pandemic and they concluded The surge in critically ill patients has meant sudden organizational changes imposed by hospital managers in order to provide an immediate response to this unprecedented human resource crisis in health care. These circumstances have fueled new nursing management challenges and opportunities that deserve our attention [18]. Researchers in Iran investigated the challenges faced by ICU nurses while providing treatment for COVID-19 patients: The nurses noted the four following obstacles while providing care for COVID-19 patients, according to a qualitative study: ‘Inefficiency of the organization in supporting nurses,’ ‘physical exhaustion,’ ‘living with uncertainty,’ and ‘psychological load of the disease,’ among others [19]. Another study in the switzerland looked at experience of middle management nurses during the covid-19 pandemic, and nurses reported the their experiences throughout the provision of care for covid-19 patients and included four macro-themes of changes, conflicting, emotions, relation [20].

## Necessity and objectives of the study

Nurses have an important role in spreading health institution directions among their colleagues and managing clinical practice nurses. Indeed, their decisions can have a significant impact on clinical nurses’ well-being and capacity to work in safe environments, as well as the overall quality of care delivered [20, 21]. Due to the high workload and limited workforce, it is essential to identify challenges related to hospitals as a front line in dealing with critically ill patients in the management of the new coronavirus (COVID-19) pandemic in the country’s medical universities. The identification of such challenges can contribute to developing a roadmap for future disaster management planning. Furthermore, capabilities, limitations, and weaknesses in providing services in the hospitals should be identified to increase the work capacity and standards, be better prepared to deal with the disasters, and reduce its devastating effects. Studying the experiences of people who were responsible in times of disaster is one of the ways to evaluate the effectiveness of the disaster management program and its strengths and weaknesses. Thus, considering the importance and specific features of this pandemic. This study aimed to explore the hospitals challenges related to covid-19 pandemic management from the iranian nurses perspective.

## Method

### Study design and setting and participants

The present study was a conventional qualitative content analysis that performed in kerman university of medical sciences from December 2021 to May 2022. Content analysis can be used to infer or deduce from qualitative or quantitative data. This strategy is beneficial when there is a lack of established theory or research literature on a particular topic [22].

This qualitative study was conducted on 35 nurses that worked in different wards of government hospitals admitted Covid-19 patients, including (management department, general wards and critical wards). Purposeful sampling strategy was used to choose the participants. 14 male nurse and 21 female nurse were interviewed in this study. The inclusion criteria consisted of the participants with a bachelor’s degree or associate degree in nursing, at least one year of work experience during in Covid-19 pandemic and willingness to participate in the study. The lack of consent to participate in the study was an exclusion criterion. Sampling continued until data saturation was reached, at which point the researcher judged that more interviews would not yield new information.

This qualitative study was conducted using in-depth and semi-structured interviews beginning with open questions, gradually followed by more detailed questions. The interviews began with broad questions such as “Please describe your experiences with Covid-19 pandemic against patients?” “What are the most important challenges and shortcomings for service providers?” “What were the causes of these challenges and shortcomings most of the time?” “What was the most common cause for these challenges?” and “Would you explain more?” Probing was also done according to the reflections of each participant about experiences of COVID-19 pandemic against nursing personnel. The interviews were taped and lasted from 25–74 min. The researcher and the nurses agreed on the time and place of the interviews. During the interviews, field notes were also obtained to accurately record and analyze the responses.

### Data analysis

The data acquired in this step was examined using Graneheim and Lundman’s method of content analysis [23]. The researcher examines the transcribed materials multiple times to fully comprehend them in this inductive approach. Then, the meaning units (words, sentences, or paragraphs) that responded to questions about pre-hospital emergency challenges and issues in handling the COVID-19 pandemic were identified, reduced, and coded. Comparable codes representing similar concepts were divided into subcategories, which were then combined to form a category (manifest level). Each category arose from a collection of related ideas, making the categories internally homogeneous yet externally heterogeneous. The key subject emerged as the interaction between the underlying meanings in categories, which express the latent meaning. To handle the coding process, a trial version of the MAX QDA 16 Software was used.

### Reliability and validity

For reliability and validity tests, this study used Lincoln and Guba’s recommendations [24]. To ensure reliability, four requirements of creditability, dependency, conformability, and transferability are required, according to this suggestion. To improve data credibility, the researchers spent 11 months interacting with data and the environment, continually making observations and compiling field notes. Peer check procedures were used to determine the data’s dependability. Monthly peer checks were conducted to ensure that the study team had a full discussion of the newly discovered data. Background information and the researchers’ own interests in the respective topics, as well as document upkeep, were used to assess data conformability. The research team, as well as other specialists and professionals in the field of qualitative research, analyzed the context of the interviews, codes, and extracted categories. The researchers were able to obtain a wide range of various comments, observations, and interpretations by using maximum variation sampling.

## Results

### The participants’ demographic data

To explore the challenges to manage COVID-19 pandemc in Iranian government hospitals, perspective of 35 nurses from hospitals different wards were studied,showen in Table 1.

**Table 1:** Demographic information of participants

Demographic Characteristics	Sub-Category	Number (Percentage Of The Sample)
Gender	Male	14(40.00)
	Female	21(60.00)
Age (Years)	25–35	13(37.14)
	36–45	12(34.28)
	≥ 46	10(28.57)
Work Experience	10 ≤	11(31.42)
	11–20	16(45.71)
	21–30	8(22.85)
Participant’s Status	Nursing Manager	5(14.28)
	Clinical Supervisor	6(17.14)
	Educational Supervisor	4(11.42)
	Nurses of General Wards	9(25.71)
	Nurses of Critical Wards (ICU, CCU And Dialysis)	11(31.42)

Critical wards: *ICU* Intensive care unit, *CCU* Coronary care unit, and dialysis unit

### Main results

**Fig. 1:**
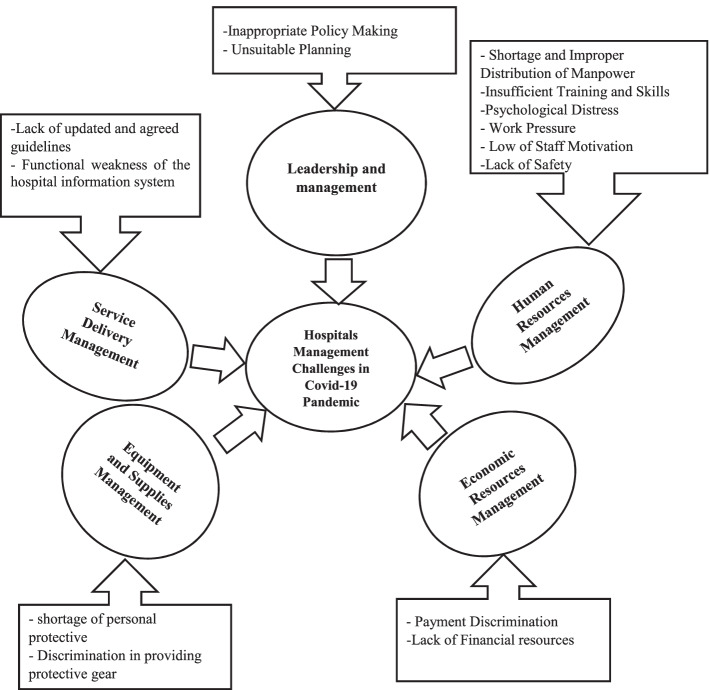
Hospitals challenges related to Covid-19 pandemic management from the Iranian nurses perspective

Based on the results of data analysis, a total of 5 categories and 14 subcategories were obtained. After several rounds of reviewing and summarizing the data and taking into account similarities and differences, five main categories were obtained through the content analysis method, including including1) Leadership and management 2) Service delivery management 3) Human resources management 4) Equipment and Supplies Management and 5) Economic resources management. as shown in Fig. 1. The five major categories were in turn classified into several subcategories extracted via analyzing hand-written notes and interviews. The categories and subcategories are described in the following sections.

### Leadership and management

The data from the interviews with the participants indicated that lack of effective management in hospitals, disregard for scientific management, managers’ arbitrary interventions, unprofessional interventions of parallel organizations, wrong policies of the ministry of health to forcibly employ medical staff, wrong policymaking for compulsory and early call for the enlistment of medical conscripts were some leadership and management challenges in confronting COVID-19 in hospitals. These challenges were divided into two categories of inappropriate policy making management and unsuitable planning.

#### Inappropriate policy making

The data in this study revealed that Appropriate participation in healthcare policy making requires an improvement in nurses’ policymaking competencies.One of the participants stated that:

*“Most of these new improvements should be implemented by nurse leaders and managers, who should also work to elevate nurses’ status in healthcare policy-making and enhance their working circumstances” (Participant 9).*

#### Unsuitable planning

Having an action plan for preparedness and response of hospitals is one of the basic principles of managing the risk of the Covid-19 pandemic. According to one of the participants:

*“There is a lack of effective planning and management communication and coordination inside and among medical institutions, as well as between superior entities like the Ministry of Health and the medical universities.” (Participant 12).*

### Service delivery management

The results of the study indicated that most of the participants considered healthcare workers confusion in providing services lack of an accurate, scientific, and integrated process in the process of examining inpatients and outpatients.These challenges were divided into lack of updated and agreed guidelines and functional weakness of the hospital information system.

#### Lack of updated and agreed guidelines

In hospitals for patient screening, contact tracing, follow-up there was no suitable process in place. Due to this, the gover and patientment hospitals became confused, and during the initial weeks, those in need did not receive proper and efficient care. One of the participants stated that:

*“If someone has the condition, there are no reliable resources that are readily available to assist them. Even healthcare professionals are absolutely unsure on where to go and what to do (Participant 7).*

*“The unwillingness of the Ministry of Health to organize physicians and specialists using a consistent diagnostic and treatment protocol for referral to medical and diagnostic centers, the great number of patients referring to take PCR test, who are often false-negative cases or a large number of patients to perform a CT test who need to undergo treatment if they are CT positive despite a negative PCR result are some important challenges” (Participant 16).*

#### Functional weakness of the hospital information system

*“The second challenge we face is the lack of effective information infrastructure or the electronic health records, as one of the challenges of our entire hospitals. Of course, the new online systems that have been created recently may help, but we do not have the electronic health system that they are talking about it in the media and we have to create a set of websites quickly to have comprehensive information in times of pandemic disease. But creating these systems is not so easy because it takes much energy and time” (Participant 3).*

### Human resources management

The data in this study revealed that the severe shortage of specialists, lack of workforce, lack of anesthetists and infectious disease specialists in some hospitals and cities, the infection of service and support staff with COVID-19 and compulsion to attend the hospital, visit of both healthy and suspected COVID-19 patients by a physician due to the shortage of physicians, lack of staff in CSR and laundry wards, lack of infectious disease specialist during the COVID-19 crisis, and lack of fixed staff in the radiology department were, inability of the Ministry of Health in organizing physicians and specialists, lack of coordination in distributing specialists, and perceiving COVID-19 to be a gender-based disease some human resource challenges in providing service during COVID-19 pandemic. these challenges were divided into 6 categories of shortage and improper distribution of manpower, insufficient training and skills,psychological distress,work pressure, lack of safety and low of staff motivation.

#### Shortage and improper distribution of manpower

Many participants reported challenges regarding their health system, especially those affecting the hospitals including improper distribution and staff shortage. One of the participants stated that:

*“The main problem faced by the university is the shortage of workforce, especially the shortage of doctors and nurses. Tehran University of Medical Sciences has always suffered from a shortage of staff, and the workforce-to-bed ratio at this university is much lower than at other universities, but this shortage is much more noticeable during this crisis this shortage. Lots of medical staff, especially formal and contractual personnel have taken their paid leave or refused to come to their hospitals pretending that they have got the disease. During the pandemic conditions, the community needs my expertise and skills. But if I shoulder off my mission, my expertise will not benefit my country and my people” (Participant 6).*

*“Due to the severe shortage of specialists in the country, physicians must visit both non-COVID-19 patients, suspected COVID-19 patients, and other patients at the same time. Furthermore, given the lack of personal protective equipment, the possibility of infecting other patients by the doctor or the possibility of physicians getting the disease from other patients is increasing. The existence of various diagnostic and therapeutic guidelines for different groups of patients has solved many of the challenges in this ward” (Participant 19).*

#### Insufficient training and skills

*“The lack of training courses due to the complexity and unknown nature of the COVID-19 crisis for senior managers and even specialists sometimes leads to disagreements or conflicting remarks about the disease. Thus, we don’t expect unskilled nursing assistants who are checking people in the street for COVID-19 using a simple thermometer to be able to respond to people’s questions or not provide irrelevant responses” (Participant 32).*

*“Most of the experienced personnel lack necessary training and skills in dealing with the COVID-19 crisis. I think this problem is rooted in the lack of training courses for the staff. We have a poor training system, and mismanagement of hospital management teams and the shortage of equipment are other problems. We were facing a severe lack of facilities at the onset of the disease but were still have the sample problem but less severely” (Participant 8).*

#### Psychological distress

*“The most important challenge for us as healthcare providers is the fear and anxiety of getting the COVID-19 disease and transmitting it to our families. In fact, most of the time we enter the house with fear and anxiety lest our family members get sick and we have to blame ourselves for the rest of our lives” (Participant 26).*

*“In general, all challenges we face in dealing with COVID-19 are due to the unknown ways of transmitting the disease and the unclear response of the immune system, fear, and anxiety. These challenges prevent the provision of effective services to COVID-19 patients” (Participant 5).*

#### Work pressure

“*The unknown course of this disease and the lack of medical staff from doctors to simple service staff have caused physical and mental fatigue. Besides, when some staff develop COVID-19 and leave the treatment team, other personnel have to work in more intensive work shifts*”.

*“One of the important problems is that the experienced staff most often tend to shoulder off their responsibility for care for patients. Thus, they take sick leave and this increases the workload of other personnel” (Participant 35).*

#### Lack of safety

*“Wards are constantly disinfected with whitewash and harmful disinfectants and this is really hurting us. The patients are also harmed and sometimes the overuse of these substances leads to serious illnesses” (Participant 14).*

*“A very important issue we are facing is non-compliance with safety protocols in medical records of COVID-19 patients, including the marking of records and the use of plastic covers to prevent the spread of the disease. Non-observance of these issues have actually led to nurses getting infected with the coronavirus” (Participant 10).*

#### Low of staff motivation

*“Depression, loss of concentration, and lack of motivation are the most important psychological consequences of the COVID-19 virus. We are really going through a terrible situation. Some of the staff even think about migrating and can no longer stand the situation” (Participant 21).*

### Equipment and supplies management

Analysis of the data from the interviews indicated that most of the participants considered lack of personal protective equipment, lack of necessary sanitary supplies for healthcare staff, lack of special medical devices in some cities, waste of time due to lack of equipment, the failure of the Deputy Minister of Health to provide disinfection equipment, lack of portable diagnostic equipment, low-pressure ventilation devices, and shortage of medication as the most important medication and technological challenges faced by nurses in dealing with the covid-19 pandemic. these challenges were divided into 2 categories of shortage of personal protective and discrimination in providing protective gear.

#### Shortage of personal protective

*“I can say that the crew had a lot of problems for the first two weeks. Alcohol, gloves, and masks were not present. These were completely absent from the market(Participant 18).*

*“Different and occasionally incompatible protocols leave healthcare employees uncertain of how to safeguard themselves” (Participant 13).*

#### Discrimination in providing protective gear

When compared to the protective equipment offered to doctors, nurses encountered severe discrimination. The dominance of the doctors and the discrimination in acquiring protective equipment were obstacles that might have caused nurses to lose interest in providing high-quality patient care.

*“Doctors are provided any type of clothing, shield, or protective equipment they desire by the nursing office because they are terrified of them, while nurses who have more interaction with patients are not given such gear” (Participant 17).*

### Economic resources management

Most of the participants pointed to discrimination in payments between physicians and other staff, irregular and delayed payments, no raising payments for outsourcing staff, and increased cost of disposable items as some financing challenges faced by hospitals during the COVID-19 pandemic. These challenges were divided into 2 categories of payment discrimination and lack of financial resources.

#### Payment discrimination

Payment discrimination particularly according to the type and degree of specialization was a subtheme in this field.According to one of the participants:

“*There is a lot of discrimination between physicians and other staff in terms of payments. Despite the high workload on other staff, our payment is much lower. In addition to being away from the family without leave and a lot of stress, we are experiencing, low payments worsen our problems*”.

*“The number of COVID-19 patients admitted and treated in the medical centers has increased tremendously in some medical centers in some provinces, and this has put a lot of pressure on the medical staff. However, despite the high workloads, payments are made very irregularly and without any specific plan. There are long delays in payments, and this has demotivated us” (Participant 15).*

#### Lack of financial resources

The average length of stay has increased, elective surgeries have been postponed and supportive facilities for staff have been provided, and the expenditures of community education have increased, all of which have reduced the profit potential of public governmental hospitals.

*“Despite receiving financial assistance from the Ministry of Health, these monies are insufficient to cover the expenditures associated with COVID-19, particularly the costs associated with personnel remuneration and Providing quality medical care” (Participant 23).*

## Discussion

The present study tried to identify challenges faced by nurses during the COVID-19 pandemic crisis. Based on the findings of the study, 5 themes and 14 sub-themes were extracted representing the challenges encountered by nurses during the COVID-19 pandemic, as discussed below:

### Category 1: leadership and management

During an pandemic, hospital leaders often face new challenges that require them to perform unusual tasks. These tasks may go beyond their previous functioning and experience. The results of the present study indicated the fact that the lack of emphasis on scientific management is one of the management and leadership challenges. Similarly, Abdi et al. (2021) considered a wide range of special managerial competencies necessary for health care leaders during the COVID-19 outbreak [3]. Sengupta et al. (2021) explored the challenges facing health care providers during the COVID-19 outbreak and concluded that identifying trained personnel, ensuring equitable staffing, and working on the motivation and morale of health care providers are essential for upgrading the health care system. In practice, however, there is a gap that is often seen as managerial incompetence and inefficiency [25]. The managerial issues identified in the present study were ineffective management in hospitals, managers’ arbitrary interventions, and lack of coordination in the distribution of physicians.

### Category 2: service delivery management

Health care guidelines and standard protocols are very important in providing health services during the COVID-19 pandemic and have raised many issues and concerns about the quality of health care services. The COVID-19 pandemic is a threat to all institutions and organizations active in the health care system. One of the service provision challenges highlighted by the participants in this study was the lack of codified clinical guidelines and the existence of parallel instructions communicated by senior authorities. Sengupta et al. (2021) also found that the knowledge, judgment, and decision of healthcare professionals have been challenged due to limited guidelines during the COVID-19 epidemic. Because there was no standard protocol or previous literature on this type of specific pandemic [25].

The disregard for professional advice and recommendations was also pointed out by the participants as one of the process-related challenges. Mirkazehi Rigi et al. (2020) also reported that the biggest challenge in dealing with COVID-19 was not taking the disease seriously by the public. One of the problems reported by the participants concerning service delivery was infrastructural issues including the lack of diagnostic and treatment facilities, the lack of adequate beds in the ward, and the lack of standard isolation rooms in the wards [1]. A similar study by Begun and Jiang (2020) also showed that the first key challenge faced by hospitals during the COVID-19 outbreak was the lack of sufficient capacity to handle the growing number of patients. In many places, the need for ICU beds and ventilators as well as staff is far beyond the maximum capacity. For example, the number of ICU patients treated in New York City health hospitals was more than three times the capacity of ICUs [1].

### Category 3: Human resources management

The findings of the present study show that “fear of COVID-19 and involvement with it” is one of the human resource challenges faced by nurses. Davoodi and Heydari (2021) also pointed out that COVID-19 has put a lot of pressure on nurses and because the COVID-19 infection progresses faster than usual in patients with underlying diseases, nurses are constantly concerned about their inability to treat patients, their safety, and that of their colleagues, and their potential risk to their health [26]. Furthermore, Rahman Razu (2021) reported that excessive work pressure causes psychological distress, insomnia, physical weakness, and also fear of infection in health care professionals [27]. Sperling (2021) also highlighted nurses’ concerns about possible infection, lack of personal protective measures, nurses’ fear of being infected with the virus or infecting others (family, friends, etc.) [28]. Another challenge related to human resources was psychological distress. Davoodi and Heydari (2021) stated that nurses have received more attention in this crisis because they play a wide range of roles at the same time: performing function roles, performing screening, caring for critically ill patients, deciding on triage protocols, and contacting families and informing them about the death of a loved one. These conditions lead to harm to the nurses. As a result, they feel unable to regulate psychologically and resist stress [26]. Accordingly, Kang (2018) revealed that the outbreak of infectious diseases causes a significant level of anxiety and fear among nurses and causes psychological harm to health care professionals [29]. Another study conducted on hospital physicians and nurses in China showed that medical care workers experienced high levels of symptoms of depression, insomnia, anxiety, and pain. Therefore, it seems that taking care of and maintaining the mental health of these employees during this critical situation is of great importance [30].

The participants in the present study identified burnout as an important barrier. Khankeh et al. (2021) also pointed to the challenge of nurses’ burnout along with constant stress, insufficient support, inability to manage human resources, and disregard for their needs. They also reported that long working hours, multiple increases in the number of patients, and high workload have reduced the mental, physical, and emotional, strength of health care workers [31]. Thus, effective management practices are needed to handle the increase in the number of hospitalized patients and the extensive workload of healthcare staff, and their frustration. Garosi et al. (2020) also showed that many psychosomatic problems have affected nurses. Physical and mental fatigue, skin problems, chronic headaches, and anxiety disorders were some of these cases, many of which account for a significant volume of studies [32].

The shortage of manpower was another challenge highlighted by the participants in this study. Labaf et al. (2020) also emphasized the shortage of specialized staff as a challenge [33]. Furthermore, Garosi et al. (2020) highlighted the lack of specialized and experienced nurses, unconventional work schedules, involuntary transfer of staff between departments, insufficient organizational support, and insufficient specific training about COVID-19 as important problems faced by nurses during the COVID-19 epidemic [32]. The participants in this study pointed to the unfair distribution of personal protective equipment. Similarly, Labaf et al. (2021) noted that the use of protective equipment is an important management challenge. In fact, there is enough equipment, but there is no control over their consumption and distribution [33].

Poor training of medical staff is also an important challenge, and neglecting this issue leads to insufficient knowledge and hinders their ability to work safely. Similar studies (e.g. Sun, 2020; Yin, 2020; Rathnayake, 2021) reported the importance of nursing education during the COVID-19 epidemic. Lack of awareness is one of the main causes of insecurity and providing training about COVID-19 prevention and control can also reduce the psychological burden and feeling of insecurity in nurses [34–36]. A study conducted by Radfar et al. (2021) on the organizational and managerial challenges experienced by nurses during the COVID-19 pandemic showed that nurses’ efforts are not appreciated by managers and organizations [12]. Accordingly, the present study highlighted employee motivation and lack of spiritual support for staff.

### Category 4: Equipment and supplies management

Concerning equipment and supplies issues, the participants stated that they were facing the problem of lack of personal protective equipment. Razu (2021) showed that incentives such as financial support, continuous monitoring, adequate protective equipment, and adequate manpower can encourage health workers to be more involved in epidemics [27]. The participants in the present study highlighted the lack of personal protective equipment as a challenge related to medical equipment and supplies. Furthermore, Rathnayake (2021) highlighted insufficient facilities and equipment such as personal protective equipment (PPE) as a challenge reported by nurses [1]. Similarly, many studies reported that inadequate resources such as personal protective equipment such as masks, disposable gloves, face shields, caps, and hand sanitizers could affect nurses’ morale and health. Thus, the contagious nature of COVID-19 can endanger the lives of nurses and their families [37, 38]. Similarly, Sengupta et al. (2021) stated that the sudden onset of COVID-19 caused an influx of essential drugs, a shortage due to a change in the conventional supply chain, and a hoarding of essential medical supplies [25].

### Category 5: Economic resoures management

Concerning financing issues, Radfar et al. (2021) stated that nurses are victims of organizational bias and highlighted the permanent financial problems and inequality in regulations [12]. Accordingly, the results of the present study highlighted the existence of pay discrimination between physicians and other staff and their irregular payments. The results of a similar study indicated that financial management is difficult for many health care professionals while they fear illness, unauthorized absence may result in reduced wages, ward requests, and job loss. Most of the respondents in this study were also concerned about financial issues and emphasized the burden of financial issues [39].

### Limitations and strengths of the research

This study has a strong point considering that it has been done in the whole country and its challenge has been extracted all over the country. However, due to the prevalence of Covid-19 disease, some interviews were not possible in person and had to be conducted virtually.

## Conclusion

Identifying the most important challenges of nursing can play an important role in improving the management of the new coronavirus pandemic (COVID-19). The analysis of the challenges by managers at local, provincial, and national levels can lead to the presentation of effective solutions to address these challenges and improve the pandemic management process in the country.

Supporting health care workers is linked to the ethical responsibility of preventing the COVID-19 disease. Insufficient protection has jeopardized the principle of ethics and the value of safety. Insufficient resources may also undermine the right to health care, especially the right of seriously ill patients in situations such as COVID-19, and the right of citizens to equal access to quality health care. It is thus advisable for the hospitals managers to ensure that adequate personal protective equipment is provided to medical care staff working on the frontline of fighting COVID-19 to ensure the safety of nurses and other staff. Creating a law to compensate for the risks that nurses face in caring for COVID-19 patients can go a long way in ensuring the interests of nurses.

### Implications for nursing management

In the COVID 19 pandemic comprehension of these challenges can assist healthcare officials to take suitable activities to resolve them, such as providing health-care facilities, supporting the health workforce, providing precise information, and performing psychological interventions on how to deal with the pandemic.

## Data Availability

The data sets generated during the current study are available from the corresponding author.
